# The effect of video-assisted discharge education after total hip replacement surgery: a randomized controlled study

**DOI:** 10.1038/s41598-022-07146-y

**Published:** 2022-02-23

**Authors:** Ozum Cetinkaya Eren, Nihal Buker, Hasan Atacan Tonak, Mustafa Urguden

**Affiliations:** 1 Physiotherapy Program, Department of Therapy and Rehabilitation, Health Services Vocational School, Alanya Alaaddin Keykubat University, Alanya, Antalya Turkey; 2 School of Physical Therapy and Rehabilitation, Pamukkale University, Denizli, Turkey; 3 Department of Physiotherapy and Rehabilitation, Faculty of Health Sciences, Akdeniz University, Antalya, Turkey; 4 Department of Orthopaedics and Traumatology, Faculty of Medicine, Akdeniz University, Antalya, Turkey

**Keywords:** Diseases, Health care

## Abstract

This study aimed to investigate the effect of a video-assisted discharge education program on activities of daily living, functionality, and patient satisfaction following total hip replacement (THR) surgery. This study included 31 patients who were randomly divided into the physiotherapy group (n = 18), and the video-assisted discharge education (VADE) group (n = 13). Both groups received a physiotherapy program. The VADE group was also received the VADE program. Face-to-face instruction was used in all of the educational programs. There was a significant difference in favor of the VADE group in Harris Hip Score, Nottingham Extended Activities of Daily Living Scale’s movement score, Tampa Scale of Kinesiophobia, Patient Satisfaction Questionnaire (*p* < 0.05). There was a significant difference between groups on resting pain levels in the first week and on resting and activity pain levels in the third month in favor of the VADE group (*p* < 0.05). The results of this study demonstrated that VADE can be effective in improving patient satisfaction and functionality, reducing pain and kinesiophobia following THR.

## Introduction

Total Hip Replacement (THR) is a common surgical procedure performed in patients who do not respond to long-term conservative treatment to reduce high pain sensation and movement limitation in the joint^1^. THR affects quality of life, patient satisfaction levels, and functional losses such as muscle weakness, muscle imbalance problems, decreased range of motion (ROM), loss of balance, length difference in lower extremities, and deterioration in walking pattern following THR, which is widely applied all over the world^2,3^. Physiotherapy and rehabilitation routinely applied to regain functional independence in daily living, restore pain and joint range of motion, eliminate muscle weakness, regulate walking pattern, prevent falls, loosening, revision surgery and dislocations in the prosthesis, increase participation in activities of daily living (ADL), quality of life (QoL) and patient satisfaction levels after THR^3,4^.

In patients undergoing THR surgery, recovery begins immediately after surgery and the recovery process continues over the years^5^. The majority of patients in this recovery process lack understanding about the activities they can do following THR surgery, and they require discharge education because they are concerned about pre-discharge pain management, movement, ADL, and support requirements^6,7^. Discharge education is a process that starts when a patient is admitted to the hospital and prepares them for treatment, care, and rehabilitation following discharge^8,9^. In addition, there are pre-operative education approaches in the literature. With multidisciplinary pre-operative education programs, people might be less anxious, may have a shorter hospital stay, and may be better able to cope with pain^3,10^. Preoperative education has been found to provide patients with a more realistic expectation of surgery and has the potential to improve patient experience and satisfaction for healthcare institutions^11,12^. Following THR, preventive rehabilitation approaches, equipment suggestions, and home settings are used to allow people to safely perform their activities of daily living at home^13^. It was determined in Kennedy et al.^14^ that patients have expectations for education on recognition of the surgical team, general knowledge about the disease and prosthesis, rehabilitation, pain management, home care activities, complications, medications, and ADL after THR surgery.

Digital health or Electronic health (eHealth) systems use modern information and communication technologies such as computers, mobile phones, wearable devices, virtual reality, and web-based applications to support the effective delivery of health services and information^15,16^. Because they are methodical and efficient, it has been recommended to employ organized training materials in which suitable movements are demonstrated through visual and aural resources^17,18^. According to Dallimore et al.^19^ the use of iPads for physiotherapy patient education for patients who have undergone hip surgery may be a more effective method of improving patient recall and satisfaction, as compared to the same content paper booklets. After hip arthroplasty micro-video education can improve the depression of patients, reduce the degree of joint pain, promote the function of hip joint, and reduce complications^20^.

As a result of demographic changes in society and patients, changes in healthcare delivery, and technological techniques, discharge education has been more effective with video aid^21^. Although few studies on video-assisted patient education have been published^22,23^, in light of our current knowledge no studies investigating the effects of video-assisted discharge education on ADL, functionality, and patient satisfaction after THR surgery have been published.

Our study aimed to investigate the effect of a video-assisted discharge education program on activities of daily living, functionality, and patient satisfaction after total hip replacement surgery.

## Methods

### Study design

All procedures performed in studies involving human participants were in accordance with the ethical standards of the institutional and/or national research committee and with the 1964 Helsinki Declaration and its later amendments or comparable ethical standards. Approval was obtained from the ethics committee of Pamukkale University, Denizli, Turkey (60116787-020/58408-04.09.2018/17). Written informed consent was obtained for experimentation from all patients and/or their legal guardian(s). The clinical trial number is NCT04774562, (01/03/2021) and it was retrospectively registered.

### Participants

Our study was performed between September 2018 and June 2019. This prospective randomized control trial comprised 31 patients (21 women, 10 men) who underwent THR surgery between the ages of 25 and 70 at Akdeniz University Hospital and a private hospital in Antalya. Patients who can understand the verbal and written information supplied to the study, who are open to conversation and cooperation and who have the same/similar type hip prosthesis were included in the study. Patients with neurological and metabolic illnesses that may cause functional impairment, patients who have already undergone prosthetic surgery from the same or opposite lower extremities, patients with mental and cognitive problems, and patients who are morbidly obese (Body Mass Index > 40 kg/m^2^) were excluded from the study. Patients who missed to attend at least one of the post-surgical evaluations for any reason, who requested to leave the study at their request and could not continue to work due to any additional discomfort developed were removed from the study. The patients were informed that the study would be terminated in case of risk of falling and any additional discomfort during the VADE or physiotherapy program. Our contact information was supplied to the patients so that they may contact us if necessary. No safety problems were encountered in the study and so was completed with the intended patients.

**Table:** 

Inclusion criteria	Exclusion criteria
To understand the verbal and written information presented to the study Being open to conversation and cooperation To have the same/similar type hip prosthesis	Have a neurological and metabolic illnesses that may cause functional impairment To have already undergone prosthetic surgery from the same or opposite lower extremities Have mental and cognitive problems, and patients who are morbidly obese (Body Mass Index > 40 kg/m^2^)

Five of the 36 patients who met the inclusion criteria were excluded from the study due to missed follow-ups or a refusal to participate. The 31 patients included in the study were randomized using systematic sampling. Based on the last number of file numbers, odd numbers were included in the physiotherapy (PT) group (n = 18) and even numbers were included in the video-assisted discharge education (VADE) group (n = 13). The block randomization method was used. In all groups, the first meeting with patients took place in the hospital room within the first five days after THR surgery. The surgeries were performed by the same surgeon in two different hospitals. Patients who had the same or similar type hip prosthesis were included in the study so that any differences that may occur were eliminated. The same physiotherapist provided physiotherapy and video-assisted discharge education to patients and their relatives without disclosing which group they were in. Due to the design of our study, blinding was not employed. VADE-physiotherapy programs and assessments had to be done by the same physiotherapist due to a lack of practicing physiotherapists.

### Physiotherapy (PT) group

The physiotherapy program given to the PT group was developed using the treatment programs applied by Can^24^ and Gray et al.^25^ after THR surgery. Breathing exercises, hip range of motion and strengthening exercises, positioning, and information about walking and ambulation were all part of the physiotherapy program. The whole program was taught practically and verbally to patients and their relatives. Information was given about the exercises to be added at the end of the first week and in the 4th week. The patients were given a physiotherapy booklet with the same content. The booklet was examined by the patient and relatives, and the same physiotherapist answered their questions. The patients were informed that they should continue the exercises for 12 weeks^24,25^.

### Video-assisted discharge education (VADE) group

In addition to the physiotherapy program delivered to the PT group, the VADE group received video-assisted discharge education on the same day by the same physiotherapist. In our study, the holistic view of physiotherapy and rehabilitation was used while organizing the VADE program. The VADE program included information about THR, preventive rehabilitation approaches, transfer activities, using stairs, self-care activities, home settings by modifying the treatment and discharge protocols of Lucas^26^ and Drummond et al.^27^. VADE was created as a written and video presentation in which a professional model demonstrates the information. The video shootings were conducted by a physiotherapist with experience in the field of physiotherapy and rehabilitation following THR surgery. When patients had questions or didn't understand something, the presentation was interrupted, and the necessary explanations were offered verbally and practically. In addition to the physiotherapy booklet, patients were given an instructional booklet that contained written and visual information developed in the same content as VADE. During VADE, the patient and relatives examined the booklet, and the same physiotherapist addressed questions.

Patient interviews were completed in approximately 30 min in the PT group and approximately 45 min in the VADE group. Since the patients were seen in the early period after the surgery and the preventative procedures and transfer methods were explained in detail, no discomfort or harm was detected in the patients except pain. For the same reason, no safety devices other than walking aids were used in the study. A healthy person was utilized to prevent harm to the hip joints of patients who had undergone surgery in photographs and videos in the VADE and booklets. In the booklets, easy-to-understand sentences were used by patients and their relatives. The booklets were printed in 11-point Arial font. Atesman's readability formula^28^ was used to design brochures.

For 12 weeks, all patients were called every 15 days and their participation in the exercises and ADL was tracked.

### Assessment methods

After physiotherapy and VADE programs, assessments were performed in the hospital room within the first 5 days following THR surgery (Descriptive Information, Pain, Patient Satisfaction Questionnaire), and at the 3-month control (Pain, Harris Hip Score (HHS), Tampa Scale of Kinesiophobia (TSK), Nottingham Extended Activities of Daily Living Scale (NEADL), Patient Satisfaction Questionnaire) in orthopedics and traumatology clinic.

The patients' descriptive information was recorded on a prepared form. The pain of patients during sleep, rest, and activity was evaluated using a visual analog scale (VAS)^29^. The HHS was used to assess the physical function of the hip. The validity and reliability of the Turkish version of the HHS were determined by Celik et al.^30^. Kinesiophobia was evaluated with TSK. The validity and reliability of the Turkish version of the TSK were determined by Tunca Yılmaz et al.^31^. ADL of the patients was evaluated with the NEADL^32^. To examine the patients' levels of satisfaction, the researchers used a five-item questionnaire. This questionnaire was used to evaluate patient satisfaction with the surgical procedure, physiotherapy program and VADE, ADL and preventative protocols, transfer activities, and home settings. Patient satisfaction levels were evaluated over 10 points with VAS.

### Statistical analysis

In the statistical analysis of data obtained in this study, the Windows-based SPSS (IBM SPSS Statistics, Version 24.0, Armonk, NY, USA) package program was used. To achieve 80% power to detect a difference with 95% confidence using the two-tailed test, a sample size of 12 participants was required for each group, including the potential drop-out^33^. Continuous variables are presented as means ± standard deviation (x ± SD) or as a median, and categorical variables as a number and percentages (%). When the parametric test assumptions were provided, Independent Sample T-Test was used to compare independent group differences; when the parametric test assumptions were not provided, Mann Whitney-U test was used to compare independent group differences. In the dependent group analyzes; when the parametric test assumptions were provided, Paired Sample T-Test was used; when the parametric test assumptions were not provided, Wilcoxon Test was used. A *p* level of ≤ 0.05 was accepted as statistically significant and interpreted^34^.

## Results

This study included 31 patients, ranging in age from 25 to 70 years old (PT Group = 55.33 ± 11.83 years, VADE Group = 49.61 ± 9.52 years). There was no significant difference in age, body mass index, daily work, and standing times between the two groups (*p* > 0.05). The other demographic characteristics of the patients are listed in Table 1.

**Table 1:** Demographic data of patients.

Variables	PT group (n = 18)	VADE group (n = 13)	*p*		
	${\overline{\text{x}}}$ ± SD	${\overline{\text{x}}}$ ± SD			
Age (years)	55.33 ± 11.83	49.61 ± 9.52	0.16^a^		
BMI (kg/m^2^)	26.86 ± 2.68	26.07 ± 2.50	0.41^a^		
Daily working times	2.00 ± 3.67	5.15 ± 5.28	0.11^b^		

Variables	PT group		VADE group		*p*
	n	%	n	%	
**Sex**					
Female	12	66.7	9	69.2	1.00^d^
Male	6	33.3	4	30.8	
**Use of cement**					
Cemented	6	33.3	3	23.1	0.69^d^
Cementless	12	66.7	10	76.9	
**Dominance on upper extremity**					
Right	16	88.9	12	92.3	1.00^d^
Left	2	11.1	1	7.7	
**Dominance on lower extremity**					
Right	12	66.7	10	76.9	0.69^d^
Left	6	33.3	3	23.1	
**Affected extremity**					
Right	8	44.4	7	53.8	0.60^c^
Left	10	55.6	6	46.2	
**Education level**					
Illiterate	3	16.7	1	7.7	–
Primary school	7	38.9	4	30.8	–
Middle school	2	11.1	1	7.7	–
High school	4	22.2	4	30.8	–
Associate degree	1	5.6	1	7.7	–
Undergraduate degree	1	5.6	2	15.4	–
**Profession**					
Retired	5	27.7	3	23.07	–
Housewife	7	38.8	3	23.07	–
Worker	2	11.1	2	15.3	–
Officer	1	5.5	1	7.6	–
Teacher	1	5.5	1	7.6	–
Farmer	2	11.1	–	–	–
Artisan	–	–	1	7.6	–
Cleaning staff	–	–	1	7.6	–
Chef	–	–	1	7.6	–

*PT* physiotherapy, *VADE* video-assisted discharge education, *BMI* body mass index, *kg* kilogram, *m* meter, *p* < 0.05 statistically significant difference, *n* sample size, $\overline{x}$ mean, *SD* standard deviation, *p*^a^ independent sample T-test, *p*^b^ Mann Whitney-U test, *p*^c^ chi-square analysis, *p*^d^ Fisher exact test.

When the pain perception levels of the patients were compared within the group, both groups showed a statistically significant decrease in sleep, rest, and activity between the first and third months (*p* < 0.05). There was a statistically significant difference between groups in resting pain levels in the first week, and, in resting and activity pain levels in the third month in favor of the VADE group (*p* < 0.05) (Table 2).

**Table 2:** Comparison of pain levels of patients.

VAS (in-group)	1st week	3rd month	*p*
	${\overline{\text{x}}}$ ± SD	${\overline{\text{x}}}$ ± SD	
**PT group**			
Sleep	5.54 ± 2.69	3.06 ± 2.28	0.008^a^*
Rest	6.97 ± 1.72	3.86 ± 2.05	0.000^c^*
Activity	8.54 ± 1.51	5.98 ± 2.24	0.000^a^*
**VADE group**			
Sleep	5.5 ± 2.01	2.09 ± 2.26	0.000^a^*
Rest	4.8 ± 2.67	2.02 ± 1.51	0.005^a^*
Activity	7.13 ± 2.42	3.78 ± 2.38	0.002^a^*

VAS (between groups)	PT group	VADE group	*p*
	Median (min–max)	Median (min–max)	
**Sleep**			
1st week	4.9 (0–10)	5.4 (2.7–9.5)	0.96^a^
3rd month	2.5 (0–8.8)	1.5 (0–7.4)	0.13^b^
**Rest**			
1st week	6.8 (4.2–10)	5 (0–8.9)	0.01^a^*
3rd month	3.45 (4–8.40)	1.8 (0–4.5)	0.01^a^*
**Activity**			
1st week	8.9 (4.6–10)	7.4 (3–10)	0.13^b^
3rd month	5.5 (2.2–10)	3.5 (0–7.5)	0.01^a^*

*PT* physiotherapy, *VAS* visual analog scale, *VADE* video-assisted discharge education, *min* minimum, *max* maximum, *p < 0.05 statistically significant difference, $\overline{x}$ mean, *SD* standard deviation, *p*^a^ paired sample T-test, *p*^b^ Mann Whitney-U test, *p*^c^: Wilcoxon test.

While there was no statistically significant difference between the groups in the HHS pain levels sub-parameter (*p* > 0.05), there was a statistically significant difference in function, absence of deformity, and ROM scores and total scores in favor of the VADE group (*p* < 0.05). When ADL levels were examined, no significant difference was found in the sub-parameters of NEADL in favor of the VADE group (*p* > 0.05), except for the movement score (*p* < 0.05). TSK results showed that the VADE group had a statistically significantly lower level of kinesiophobia than the PT group (*p* < 0.05) (Table 3). When patient satisfaction levels were examined, there was a statistically significant difference between groups on scores for all parameters in the first week and third month, with the VADE group having significantly higher scores than the PT group (*p* < 0.05) (Table 4).

**Table 3:** Comparison of hip function, activities of daily living and kinesiophobia.

HHS	PT group		VADE group		*p*
	${\overline{\text{x}}}$ ± SD	Median (min–max)	${\overline{\text{x}}}$ ± SD	Median (min–max)	
Pain	28.44 ± 13.38	30 (10–44)	35.84 ± 8.84	40 (20–44)	0.135^b^
Function	29.22 ± 10.22	29.5 (11–47)	40.07 ± 5.80	39 (29–47)	0.002^a^*
Absence of deformity	1.50 ± 1.20	1 (0–4)	3.15 ± 0.80	3 (2–4)	0.000^b^*
ROM	4.57 ± 0.62	4.8 (2.5–5)	4.92 ± 0.10	4.95 (4.69–5)	0.034^b^*
Total	63.74 ± 23.49	62.72 (23.5–98)	83.99 ± 12.46	81.8 (66.85–100)	0.004^b^*

NEADL	PT group	VADE group	*p*		
	Median (min–max)	Median (min–max)			
Movement	13 (5–18)	16 (7–18)	0.038^b^*		
Kitchen Activities	12 (3–15)	15 (7–15)	0.106^b^		
Housework	11 (2–15)	13 (9–15)	0.146^b^		
Leisure-time Activities	12 (5–18)	15 (4–18)	0.352^b^		
Total	47.5 (17–66)	59 (41.64)	0.170^b^		

TSK	PT group	VADE group	*p*		
	${\overline{\text{x}}}$ ± SD	${\overline{\text{x}}}$ ± SD			
Total	48.05 ± 6.59	39.38 ± 8.13	0.003^a^*		

*PT* physiotherapy, *VADE* video-assisted discharge education, *ROM* range of motion, *HHS* Harris Hip Score, *NEADL* Nottingham Extended Activities of Daily Living Scale, *TSK* Tampa Scale of Kinesiophobia, *min* minimum, *max* maximum, *p < 0.05 statistically significant difference, $\overline{x}$ mean, *SD* standard deviation, *p*^a^ independent sample T-test, *p*^b^ Mann Whitney-U test.

**Table 4:** Comparison of patient satisfaction levels.

Patient satisfaction questionnaire (in-group)	1st week	3rd month	*p*
	${\overline{\text{x}}}$ ± SD	${\overline{\text{x}}}$ ± SD	
**PT group**			
Surgical procedure	5.15 ± 2.56	5.77 ± 2.29	0.123^a^
Physiotherapy or VADE program	6.38 ± 2.93	6.87 ± 2.46	0.273^a^
ADL and prevention protocols	6.06 ± 2.66	7.01 ± 2.34	0.028^a^*
Transfer activities	6.67 ± 2.20	6.85 ± 2.24	0.705^a^
Home settings	6.17 ± 2.16	6.62 ± 2.12	0.201^a^
**VADE group**			
Surgical procedure	8.35 ± 1.51	8.62 ± 1.12	0.615^a^
Physiotherapy or VADE program	9.12 ± 0.89	9.46 ± 0.64	0.279^a^
ADL and prevention protocols	8.96 ± 1.34	9.48 ± 0.52	0.251^a^
Transfer activities	9.13 ± 1.06	9.72 ± 0.36	0.091^c^
Home settings	8.57 ± 1.95	9.52 ± 0.55	0.068^c^

Patient satisfaction questionnaire (between groups)	PT group	VADE group	*p*
	Median (min–max)	Median (min–max)	
**Surgical procedure**			
1st week	5.6 (0.4–8.9)	8.5 (4.7–10)	0.000^a^*
3rd month	5.7 (1.2–8.5)	8.6 (6.8–10)	0.000^a^*
**Physiotherapy or VADE program**			
1st week	7.2 (0–10)	9.3 (7.2–10)	0.001^a^*
3rd month	7.2 (2.3–10)	9.6 (8.2–10)	0.001^b^*
**ADL and prevention protocols**			
1st week	6.2 (0–10)	9.6 (6–10)	0.000^b^*
3rd month	7.3 (2.8–10)	9.5 (8.5–10)	0.001^b^*
**Transfer activities**			
1st week	6.5 (3.3–10)	9.5 (6.3–10)	0.001^b^*
3rd month	6.95 (2.4–10)	10 (9–10)	0.000^b^*
**Home settings**			
1st week	63.5 (26–96)	92 (31–100)	0.001^b^*
3rd month	71 (30–96)	97 (83–100)	0.000^b^*

*PT* physiotherapy, *VADE* video-assisted discharge education, *min* minimum, *max* maximum, *ADL* activities of daily living, *p < 0.05 statistically significant difference, $\overline{x}$ mean, *SD* standard deviation, *p*^a^ paired sample T-test, *p*^b^ Mann Whitney-U test, *p*^c^: Wilcoxon test.

## Discussion

Our study planned to investigate the effect of video-assisted discharge education program and physiotherapy program given to the patient and their relatives after THR on activities of daily living, functionality, and patient satisfaction and to reveal the difference between them. VADE was found to reduce pain perception and kinesiophobia, improve hip function, and increase patient satisfaction. The VADE and PT groups had similar ADL outcomes.

According to the current literature, patient education prior to THR surgery reduces anxiety, postoperative discomfort, and hospital stay, improves functionality, and increases patient satisfaction, and it is recommended that the variety of patient education and materials be increased^14,35,36^. According to Edwards et al.^37^, education in hip or knee replacement can be delivered in a variety of ways, including verbal communication, group training, video, or a booklet. Patients' knowledge and abilities concerning orthopedic surgery can be improved through face-to-face, web-based, audio, or video-assisted educations^17^. Patients gain a better understanding through face-to-face teaching since they have the opportunity to ask questions. Therefore, when combined with face-to-face instruction with a healthcare worker, video-assisted educations become more effective^17,37^. In our study, we delivered video-assisted discharge education to patients and their relatives in the form of face-to-face education after THR surgery. In addition, all patients in the PT and VADE groups received visual and written booklets with pertinent information following their treatment and education.

Previous research has found that pain persists for the first six months^38^ and that patients need more pain management education following joint replacements^14^. Young and Buvanendran^39^ reported that pain treatment following THR is dependent on factors such as length of hospital stay, early mobilization, exercise, effective analgesic use, and functional discharge criteria. In our study, both groups had a reduction in pain perception. However, we believe that the VADE group's functional discharge education, which includes teaching patients about hip precautions and proper activity angles, contributes to a lower impression of pain during rest and activity.

The main purpose of rehabilitation in the acute phase after THR surgery is to prevent complications and maximize function by mobilizing the patient^40^. In addition, it was reported that not all patients showed similar functional improvement after THR and 30% of patients were functionally limited in the 2 years after surgery^41^. In a previous study followed by video-assisted interviews and booklets about nursing care and the use of assistive devices following THR surgery, it was discovered that patients in the education group have less hospitalization period and had better functional outcomes^42^. In our study, similar to the literature, the functionality results of the HHS were higher in the VADE group. Because of including hip precaution protocols, transfer and use of stair activities, self-care activities, self-help tools, car use, and home settings, it suggests that the VADE program can be effective in improving functionality.

Pain and functional problems may affect the patient's participation in ADL after THR. Compared with the usual discharge and rehabilitation procedures advanced postoperative training involving more functional and early rehabilitation is more effective in ensuring participation in ADL after THR surgery^43^. In our study, only the NEADL mobility scores sub-parameter indicated a significant difference in favor of the VADE group. We believe that the lack of a significant difference in the kitchen, housework, and leisure-time activities sub-parameters is attributable to the fact that the evaluations were conducted at an early time of 3 months, which could be due to the early time for participation in these sub-parameters.

After THR surgery, patients with high anxiety and a history of falling were shown to experience fear of falling and kinesiophobia^2,44^. Kinesiophobia has also been connected to a reduction in ADL participation and the avoidance of physical activities^45^. According to a previous study, consistent regular participation in physiotherapy and patient education was the reason ​for the low occurrence of kinesiophobia in THR patients^46^. In our study, it was found that the VADE program significantly reduced kinesiophobia compared to the physiotherapy program which is consistent with the literature. It is possible to say that the fear-avoidance, fear of movement, and re-injury are all reduced in the VADE group because we educate the facts that patients will use in their daily lives with detailed and explanatory information.

Patient satisfaction is an important parameter in orthopedic surgery^47^. According to Neuprez et al.^48^, patient satisfaction can be improved by determining and achieving expectations in hip and knee arthroplasty. THR patients who get education have lower anxiety, pain, and length of hospital stays, as well as higher patient satisfaction^14,36^. Our findings corroborate previous studies. After THR surgery VADE, which includes information to avoid and attention after surgery, stair climbing, transfer and self-care activities, car transfer, and home settings, was found to have a positive effect on patient satisfaction in the parameters of adaptation to ADL, hip precaution protocols, transfer activities, and home settings.

Our study has some strengths and limitations. The strengths of our study are the use of face-to-face video-assisted discharge education and education booklets that were clearly and understandably designed, providing the necessary assistance by calling the patients on a regular basis, and offering education to patients' relatives in both groups. Additionally, there is no study in the existing literature that examines the effects of VADE on ADL, functionality, and patient satisfaction in THR patients after surgery. Limitations of our study can be said as the patients were not seen before surgery and the long-term results were not evaluated.

## Conclusion

Our study showed that video-assisted discharge education and education booklets prepared in accordance with THR patients reduced the patients' pain levels and kinesiophobia and increased the functionality and patient satisfaction. Further research is needed to investigate the long-term results of VADE in THR patients. We believe that the use of video-assisted discharge education and education booklets should be made routine in order to make positive contributions to patients undergoing total hip replacement surgery in clinics.
